# Supplementary material for: Social network centrality predicts dietary decisions in a wild bird population
Source: iScience. 2024 Mar 26;27(5):109581. doi: 10.1016/j.isci.2024.109581 (PMC11024920; doi:10.1016/j.isci.2024.109581)
Supplement: Document S1. Figures S1 and S2 and Tables S1–S10 [file mmc1.pdf]

iScience, Volume *27*

## Supplemental information

# Social network centrality predicts dietary decisions in a wild bird population

**Keith McMahon, Nicola M. Marples, Lewis G. Spurgin, Hannah M. Rowland, Ben C. Sheldon, and Josh A. Firth**

# Supplementary Information: Social network centrality shapes dietary decisions in a wild bird population

Keith McMahon[1]*, Nicola M. Marples[2], Lewis G. Spurgin[3], Hannah M. Rowland[4,5], Ben C. Sheldon[1] & Josh A. Firth[1,6]*

[1]Department of Biology, Oxford University, Oxford, UK

[2]Department of Zoology, School of Natural Sciences, Trinity College Dublin, Eire

[3]School of Biological Sciences, University of East Anglia, Norwich, UK

[4]Max Planck Institute for Chemical Ecology, Jena, Germany

[5]Department of Zoology, University of Cambridge, Cambridge, UK

[6]School of Biology, University of Leeds, Leeds, UK

*Correspondence: Keith.McMahon@biology.ox.ac.uk & Joshua.Firth@biology.ox.ac.uk

**SUPPLEMENTARY INFORMATION CONTENT:**

**(1) Supplementary Figures**

**-Figure S1. Social centrality metrics and novel food usage**

**-Figure S2. Basic social measures and novel food usage**

**(2) Supplementary Tables**

**-Table S1. Summary of experimental procedure**

**-Table S2. Full model output: Social network strength and novel food usage**

**-Table S3. Full model output: Average edge weight and novel food usage**

**-Table S4. Full model output: Eigenvector centrality and novel food**

**-Table S5. Full model output: Mean gathering event size and novel food usage**

**-Table S6. Full model output: Unique flockmates and novel food usage**

**-Table S7. Full model output: Social network strength and first feeder used**

**-Table S8. Full model output: Social network strength and time delay to use novel food**

**-Table S9. Full model output: Social network strength and overall time delay to use novel food**

**-Table S10. Full model output: Social network strength and exploitation**

**(3) Supplementary Methods S1 Procedure of dyeing peanut granules, related to STAR METHODS.**

The green dye for the food was prepared by mixing O'Brien's (Citywest, Dublin 24, Ireland) liquid green 90 food colouring in the ratio of 5 ml dye to 500 ml water. This solution was then mixed with 500 g of kibbled peanut. The mixture was placed in an oven at 50°C for 20–30 min until dry. This was repeated with O’Brien’s Christmas Red for the red dyed peanut.

## SUPPLEMENTARY FIGURES

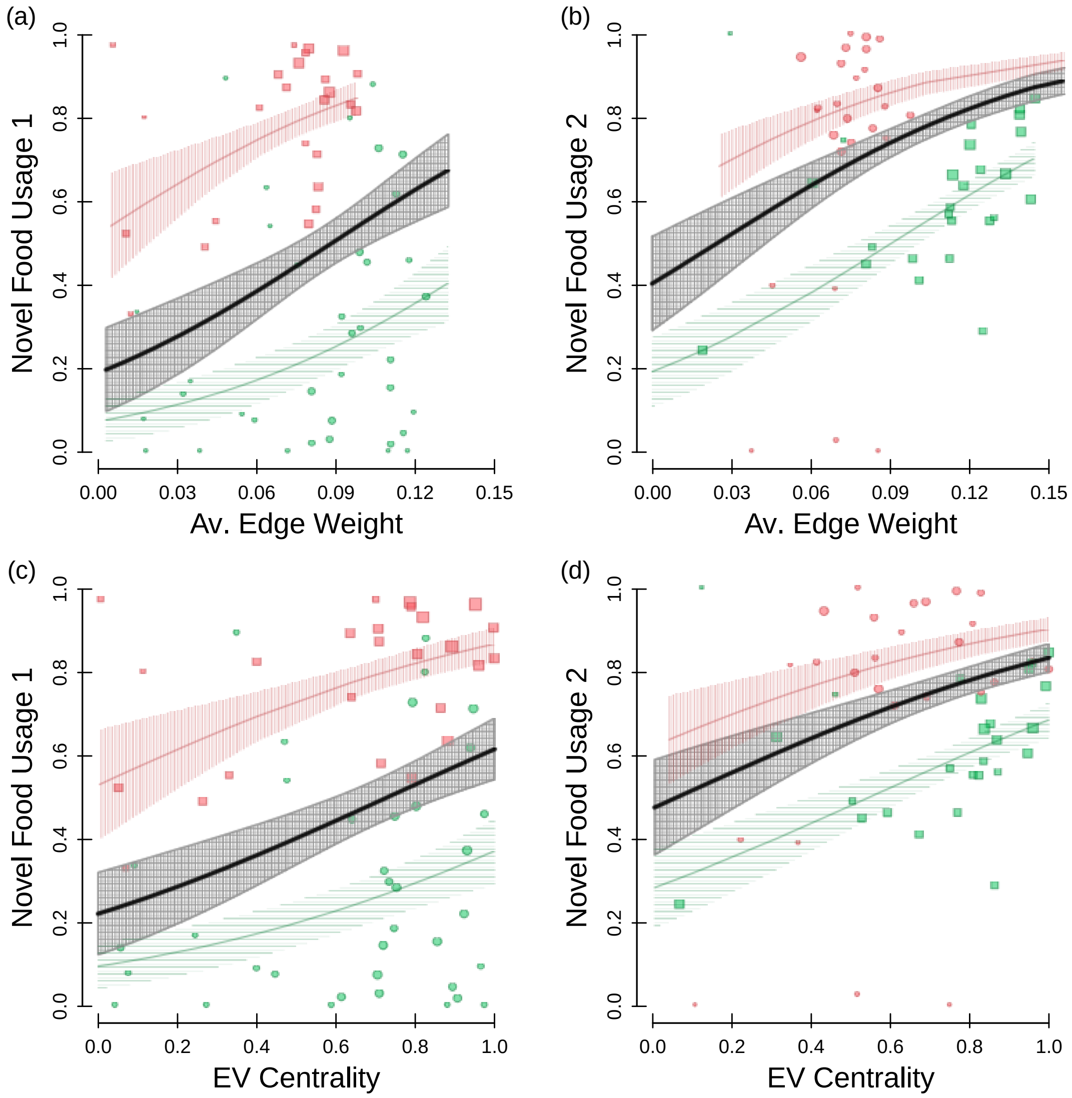


**Figure S1: Social centrality metrics and novel food usage, related to Figure 2**. Prior social centrality (x axis), as measured as (a-b) Average edge weight and (c-d) eigenvector centrality, and subsequent novel food usage (proportion of novel food usage – y axis) for the (a,c) first trial, and (b,d) second trial. The point positions show the individual data points, point colour shows the colour of the novel food (red or green dyed peanut), point shape shows which experimental site the individual was at (site 1 or site 2), and point size indicates weight of the data point i.e. the total number of detections (at both the novel, and familiar food feeder). The lines show the GLM fit, and the surrounding polygons show the associated standard error around this estimate, with the red lines showing the fit for the red novel food site, the green line showing the fit for the green novel food site, and the black line denoting the overall fit. See Table S3 & S4 for full model details.

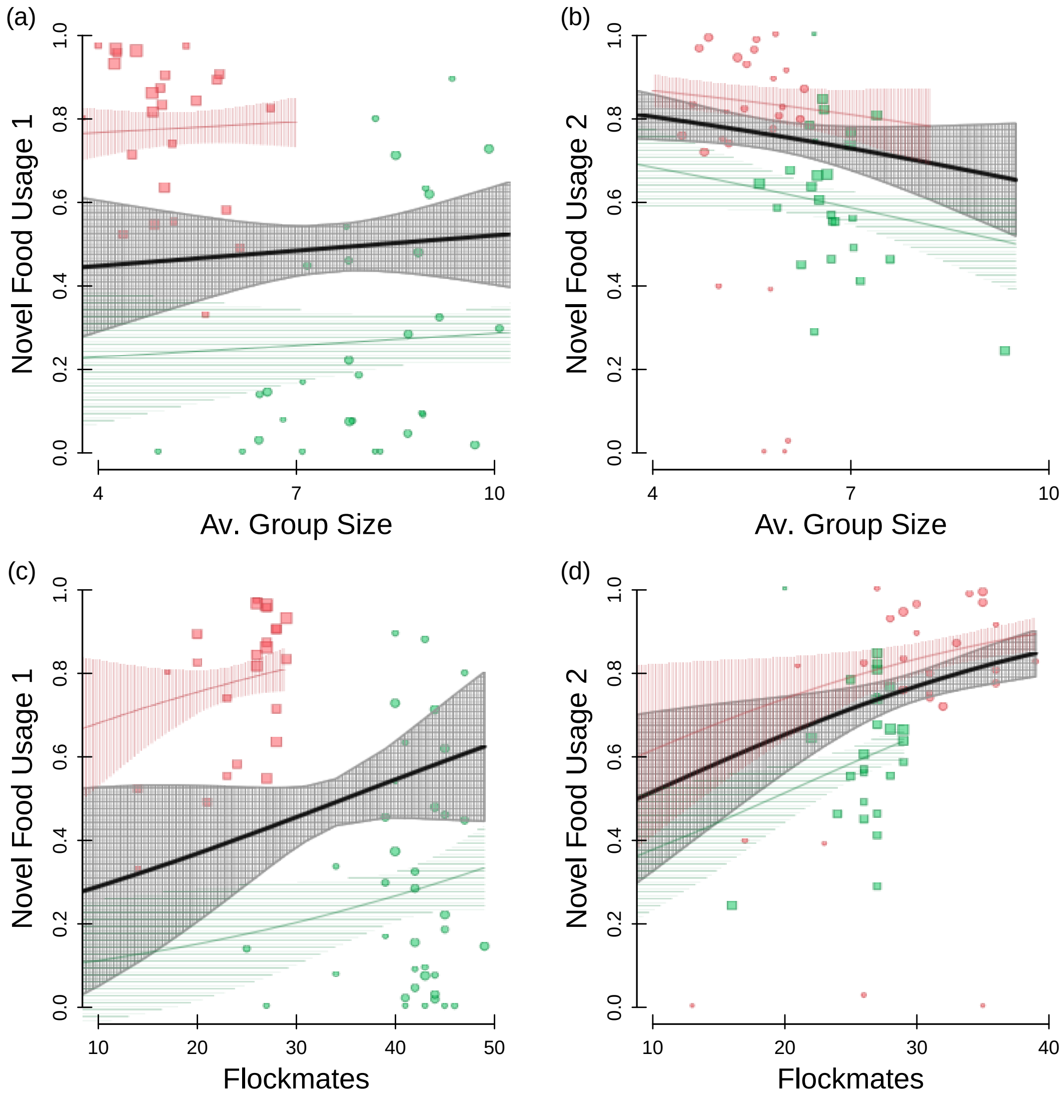


**Figure S2: Basic social measures and novel food usage, related to Figure 2**. Average group size is the average size of the flocking event that the individual was observed in, and flockmates is the total number of unique individuals the individual was observed occurring with in at least one flocking event. Prior basic measures (x axis), as measured as (a-b) Average group size and (c-d) number of flockmates, and subsequent novel food usage (proportion of novel food usage – y axis) for the (a,c) first trial, and (b,d) second trial. The point positions show the individual data points, point colour shows the colour of the novel food (red or green dyed peanut), point shape shows which experimental site the individual was at (site 1 or site 2), and point size indicates weight of the data point i.e. the total number of detections (at both the novel, and familiar food feeder). The lines show the GLM fit, and the surrounding polygons show the associated standard error around this estimate, with the red lines showing the fit for the red novel food site, the green line showing the fit for the green novel food site, and the black line denoting the overall fit. See Table S5 & S6 for full model details.

## SUPPLEMENTARY TABLES

**Table S1: Summary of experimental procedure, related to Figure 1.** The study protocol at each of the sites, showing the phase of the study and food-types used over the data-collection days and the fine-scaling positioning of the feeders within the feeding sites.

| Site | Phase | Day | Food Type | Position |
|---|---|---|---|---|
| 1 | Baseline | 1-12 | Familiar | Mid |
| | Trial 1 | 13-14 | Familiar | Side 1 |
| | | | Green | Side 2 |
| | | 15-16 | Familiar | Side 2 |
| | | | Green | Side 1 |
| | Trial 2 | 16-17 | Familiar | Side 2 |
| | | | Red | Side 1 |
| | | 18-19 | Familiar | Side 1 |
| | | | Red | Side 2 |
| 2 | Baseline | 1-12 | Familiar | Mid |
| | Trial 1 | 13-14 | Familiar | Side 2 |
| | | | Red | Side 1 |
| | | 15-16 | Familiar | Side 1 |
| | | | Red | Side 2 |
| | Trial 2 | 16-17 | Familiar | Side 2 |
| | | | Green | Side 1 |
| | | 18-19 | Familiar | Side 1 |
| | | | Green | Side 2 |

**Table S2: Social network strength and novel food usage model outputs, related to Figure 2.** Output of GLMs assessing the relationship between individuals' propensity to use novel food (response variable) and individuals' prior network strength (Figure 2 - Main Text), along with the other fitted explanatory variables. Each column holds the test statistics for (A) experimental trial 1 and (B) experimental trial 2. Each row gives the result for each explanatory variable, with 'Sex' in relation to female birds, Age in relation to adult birds, Immigrant status in relation to residents, and the 'Strength' as weighted network degree directly prior to each experimental trial (see Methods) and 'Observations' as the number of records.

| | (A) Experimental Trial 1 | | | | | (B) Experimental Trial 2 | | | | |
|---|---|---|---|---|---|---|---|---|---|---|
| | Coeff. | SE | T | P | $P_{rand}$ | Coeff. | SE | T | P | $P_{rand}$ |
| *Intercept* | -3.4250 | 0.9927 | -3.4502 | 0.0011 | 0.001 | 0.3557 | 0.4499 | 0.7908 | 0.4333 | 0.001 |
| *Sex (Male)* | 0.2716 | 0.2789 | 0.9736 | 0.3346 | 0.530 | 0.2671 | 0.2011 | 1.3281 | 0.191 | 0.396 |
| *Sex (Unk)* | -0.2357 | 1.0475 | -0.225 | 0.8228 | 0.752 | -0.1956 | 0.3889 | -0.503 | 0.6175 | 0.772 |
| *Age (Juv)* | 0.4943 | 0.2612 | 1.8928 | 0.0637 | 0.242 | 0.2943 | 0.1902 | 1.5469 | 0.1291 | 0.366 |
| *Immigrant* | 0.2481 | 0.3388 | 0.7322 | 0.4672 | 0.656 | 0.2103 | 0.2233 | 0.9418 | 0.3514 | 0.546 |
| *Site* | 3.4020 | 0.7086 | 4.801 | 0.0001 | 0.016 | -1.592 | 0.2672 | -5.9592 | 0.0001 | 0.150 |
| *Strength* | 0.5285 | 0.2347 | 2.2517 | 0.0284 | 0.010 | 0.4668 | 0.1500 | 3.1114 | 0.0033 | 0.012 |
| *Observations* | 0.0000 | 0.0001 | -0.091 | 0.9278 | 0.972 | 0.0001 | 0.0001 | -0.087 | 0.9310 | 0.968 |

**Table S3: Average edge weight and novel food usage model outputs, related to Figure 2 and Figure S1.** Output of GLMs assessing the relationship between individuals' propensity to use novel food (response variable) and individuals' average edge weight (Figure S1a;S1b), along with the other fitted explanatory variables. Each column holds the test statistics for (A) experimental trial 1 and (B) experimental trial 2. Each row gives the result for each explanatory variable, with 'Sex' in relation to female birds, Age in relation to adult birds, Immigrant status in relation to residents, and the 'Edge' as average non-zero edge weight directly prior to each experimental trial (see Methods) and 'Observations' as the number of records.

| | (A) Experimental Trial 1 | | | | | (B) Experimental Trial 2 | | | | |
|---|---|---|---|---|---|---|---|---|---|---|
| | Coeff. | SE | T | P | $P_{rand}$ | Coeff. | SE | T | P | $P_{rand}$ |
| *Intercept* | -2.8021 | 0.7216 | -3.8833 | 0.0001 | 0.0001 | 0.2123 | 0.4475 | 0.4745 | 0.6375 | 0.002 |
| *Sex (Male)* | 0.2257 | 0.2763 | 0.817 | 0.4175 | 0.642 | 0.2974 | 0.1978 | 1.5033 | 0.1399 | 0.348 |
| *Sex (Unk)* | -0.446 | 1.0028 | -0.4447 | 0.6583 | 0.532 | -0.0812 | 0.3914 | -0.2074 | 0.8367 | 0.922 |
| *Age (Juv)* | 0.4891 | 0.261 | 1.8738 | 0.0664 | 0.25 | 0.3282 | 0.1876 | 1.7499 | 0.0871 | 0.302 |
| *Immigrant* | 0.1774 | 0.3361 | 0.5279 | 0.5997 | 0.754 | 0.2717 | 0.2216 | 1.2261 | 0.2267 | 0.436 |
| *Site* | 2.55 | 0.3977 | 6.4116 | 0.0001 | 0.236 | -1.8735 | 0.2753 | -6.8054 | 0.0001 | 0.006 |
| *Edge* | 16.4625 | 7.2095 | 2.2835 | 0.0264 | 0.022 | 15.9091 | 4.5385 | 3.5053 | 0.0011 | 0.004 |
| *Observations* | 0.0001 | 0.0001 | 0.18 | 0.8578 | 0.91 | 0.0001 | 1E-04 | -0.2065 | 0.8374 | 0.928 |

**Table S4: Eigenvector centrality and novel food usage model outputs, related to Figure 2 and Figure S1.** Output of GLMs assessing the relationship between individuals' propensity to use novel food (response variable) and individuals' eigenvector centrality (Figure S1c;S1d), along with the other fitted explanatory variables. Each column holds the test statistics for (A) experimental trial 1 and (B) experimental trial 2. Each row gives the result for each explanatory variable, with 'Sex' in relation to female birds, Age in relation to adult birds, Immigrant status in relation to residents, and the 'Eigenvector' as weighted eigenvector centrality directly prior to each experimental trial (see Methods) and 'Observations' as the number of records.

| | (A) Experimental Trial 1 | | | | | (B) Experimental Trial 2 | | | | |
|---|---|---|---|---|---|---|---|---|---|---|
| | Coeff. | SE | T | P | $P_{rand}$ | Coeff. | SE | T | P | $P_{rand}$ |
| *Intercept* | -2.4432 | 0.5825 | -4.1941 | 0.0001 | 0.001 | 0.3543 | 0.4713 | 0.7517 | 0.4562 | 0.001 |
| *Sex (Male)* | 0.2578 | 0.2787 | 0.9249 | 0.3591 | 0.554 | 0.2684 | 0.2051 | 1.3089 | 0.1973 | 0.396 |
| *Sex (Unk)* | -0.3282 | 1.0023 | -0.3274 | 0.7446 | 0.652 | -0.2009 | 0.3947 | -0.5091 | 0.6132 | 0.758 |
| *Age (Juv)* | 0.5059 | 0.2616 | 1.934 | 0.0584 | 0.226 | 0.2866 | 0.1923 | 1.4901 | 0.1433 | 0.384 |
| *Immigrant* | 0.2423 | 0.3395 | 0.7138 | 0.4784 | 0.656 | 0.2187 | 0.2278 | 0.9598 | 0.3424 | 0.538 |
| *Site* | 2.3106 | 0.3414 | 6.7671 | 0.0001 | 0.718 | -1.4989 | 0.2714 | -5.5235 | 0.0001 | 0.302 |
| *Eigenvector* | 1.7357 | 0.7585 | 2.2881 | 0.0261 | 0.05 | 1.7099 | 0.5834 | 2.9307 | 0.0053 | 0.012 |
| *Observations* | 0.0000 | 0.0001 | -0.3856 | 0.7013 | 0.818 | 0.0000 | 1E-04 | -0.0523 | 0.9585 | 0.988 |

**Table S5: Mean gathering event size and novel food usage model outputs, related to Figure 2 and Figure S2.** Output of GLMs assessing the relationship between individuals' propensity to use novel food (response variable) and individuals' average flock size (Figure S2a;S2b), along with the other fitted explanatory variables. Each column holds the test statistics for (A) experimental trial 1 and (B) experimental trial 2. Each row gives the result for each explanatory variable, with 'Sex' in relation to female birds, Age in relation to adult birds, Immigrant status in relation to residents, and the 'Flock size' as mean number of individuals within each flocking event the individual was observed in directly prior to each experimental trial (see Methods) and 'Observations' as the number of records.

| | (A) Experimental Trial 1 | | | | | (B) Experimental Trial 2 | | | | |
|---|---|---|---|---|---|---|---|---|---|---|
| | Coeff. | SE | T | P | $P_{rand}$ | Coeff. | SE | T | P | $P_{rand}$ |
| *Intercept* | -1.7901 | 1.6104 | -1.1116 | 0.2712 | 0.278 | 2.2743 | 0.8448 | 2.692 | 0.01 | 0.422 |
| *Sex (Male)* | 0.1825 | 0.2921 | 0.6245 | 0.5349 | 0.704 | 0.0399 | 0.2023 | 0.1973 | 0.8445 | 0.904 |
| *Sex (Unk)* | -0.4471 | 0.9903 | -0.4515 | 0.6535 | 0.532 | -0.476 | 0.4422 | -1.0765 | 0.2876 | 0.412 |
| *Age (Juv)* | 0.5458 | 0.2779 | 1.9639 | 0.0547 | 0.202 | 0.1677 | 0.2003 | 0.8371 | 0.4071 | 0.606 |
| *Immigrant* | 0.1904 | 0.3613 | 0.527 | 0.6003 | 0.732 | -0.0225 | 0.2284 | -0.0986 | 0.9219 | 0.930 |
| *Site* | 2.1875 | 0.6794 | 3.2195 | 0.0022 | 0.930 | -1.3842 | 0.3668 | -3.7738 | 0.0001 | 0.534 |
| *Flock Size* | 0.0487 | 0.1684 | 0.2895 | 0.7733 | 0.676 | -0.1404 | 0.1526 | -0.9199 | 0.3627 | 0.232 |
| *Observations* | 0.0001 | 0.0001 | 1.1048 | 0.2741 | 0.322 | 0.0000 | 0.0001 | 2.3104 | 0.0256 | 0.134 |

**Table S6: Unique flockmates and novel food usage model outputs, related to Figure 2 and Figure S2.** Output of GLMs assessing the relationship between individuals' propensity to use novel food (response variable) and their number of unique flockmates (Figure S2c;S2d), along with the other fitted explanatory variables. Each column holds the test statistics for (A) experimental trial 1 and (B) experimental trial 2. Each row gives the result for each explanatory variable, with 'Sex' in relation to female birds, Age in relation to adult birds, Immigrant status in relation to residents, and the 'Flockmates' as sum of the number of unique individuals seen in the same flocking events as themselves directly prior to each experimental trial (see Methods) and 'Observations' as the number of records.

| | (A) Experimental Trial 1 | | | | | (B) Experimental Trial 2 | | | | |
|---|---|---|---|---|---|---|---|---|---|---|
| | Coeff. | SE | T | P | $P_{rand}$ | Coeff. | SE | T | P | $P_{rand}$ |
| *Intercept* | -2.7682 | 1.9309 | -1.4337 | 0.1574 | 0.028 | -0.2265 | 1.2268 | -0.1846 | 0.8544 | 0.001 |
| *Sex (Male)* | 0.2176 | 0.2875 | 0.7568 | 0.4525 | 0.628 | 0.1025 | 0.2014 | 0.5089 | 0.6134 | 0.732 |
| *Sex (Unk)* | -0.3795 | 1.0127 | -0.3747 | 0.7093 | 0.596 | -0.4656 | 0.4018 | -1.1587 | 0.2528 | 0.416 |
| *Age (Juv)* | 0.5626 | 0.2698 | 2.0855 | 0.0418 | 0.184 | 0.1811 | 0.1966 | 0.9212 | 0.362 | 0.576 |
| *Immigrant* | 0.3196 | 0.3807 | 0.8396 | 0.4048 | 0.564 | 0.0231 | 0.2252 | 0.1025 | 0.9188 | 0.960 |
| *Site* | 2.6957 | 0.9638 | 2.7968 | 0.0071 | 0.344 | -1.1998 | 0.3843 | -3.1216 | 0.0032 | 0.874 |
| *Flockmates* | 0.0361 | 0.0479 | 0.7535 | 0.4544 | 0.180 | 0.0565 | 0.0385 | 1.4674 | 0.1494 | 0.100 |
| *Observations* | 0.0000 | 0.0001 | 0.3305 | 0.7423 | 0.740 | 0.0000 | 0.0001 | 1.4246 | 0.1613 | 0.326 |

**Table S7: Social network strength and first feeder used model outputs, related to Figure 3.** Output of GLMs assessing the relationship between whether individuals are first detected on the novel food feeder when they first arrive at the experimental trial and their prior network strength (Figure 3 - Main Text), along with the other fitted explanatory variables. Each column holds the test statistics for (A) experimental trial 1 and (B) experimental trial 2. Each row gives the result for each explanatory variable, with 'Sex' in relation to female birds, Age in relation to adult birds, Immigrant status in relation to residents, and the 'Strength' as weighted network degree directly prior to each experimental trial (see Methods) and 'Observations' as the number of records.

| | (A) Experimental Trial 1 | | | | | (B) Experimental Trial 2 | | | | |
|---|---|---|---|---|---|---|---|---|---|---|
| | Coeff. | SE | T | P | $P_{rand}$ | Coeff. | SE | T | P | $P_{rand}$ |
| *Intercept* | -0.667 | 1.2864 | -0.5185 | 0.6064 | 0.85 | 1.1073 | 1.3439 | 0.824 | 0.4146 | 0.074 |
| *Sex (Male)* | 0.4993 | 0.8285 | 0.6026 | 0.5496 | 0.51 | -0.7047 | 0.7176 | -0.982 | 0.3317 | 0.402 |
| *Sex (Unk)* | 1.2155 | 1.9456 | 0.6247 | 0.535 | 0.378 | -19.37 | 2168.40 | -0.0089 | 0.9929 | 0.006 |
| *Age (Juv)* | -0.0508 | 0.8543 | -0.0595 | 0.9528 | 0.98 | 0.6231 | 0.7775 | 0.8015 | 0.4274 | 0.448 |
| *Immigrant* | 0.892 | 1.1421 | 0.781 | 0.4386 | 0.386 | -1.4231 | 1.1392 | -1.2492 | 0.2185 | 0.196 |
| *Site* | -0.4084 | 1.1975 | -0.341 | 0.7345 | 0.164 | 1.2332 | 0.9898 | 1.246 | 0.2197 | 0.088 |
| *Strength* | 0.2896 | 0.413 | 0.7012 | 0.4865 | 0.29 | -0.5507 | 0.5742 | -0.9592 | 0.3429 | 0.284 |
| *Observations* | 0.0001 | 0.0001 | -1.9471 | 0.0573 | 0.012 | 0.0000 | 0.0001 | -0.6424 | 0.5241 | 0.526 |

**Table S8: Social network strength and time delay to use novel food, related to Figure 3.** Output of LMs assessing the relationship between the amount of time taken for each individual to first land on the feeding perch of the novel food (quantified as time of day they were first recorded on the novel food), and their prior network strength, along with the other fitted explanatory variables. Each column holds the test statistics for (A) experimental trial 1 and (B) experimental trial 2. Each row gives the result for each explanatory variable, with 'Sex' in relation to female birds, Age in relation to adult birds, Immigrant status in relation to residents, and the 'Strength' as weighted network degree directly prior to each experimental trial (see Methods) and 'Observations' as the number of records.

| | (A) Experimental Trial 1 | | | | | (B) Experimental Trial 2 | | | | |
|---|---|---|---|---|---|---|---|---|---|---|
| | Coeff. | SE | T | P | $P_{rand}$ | Coeff. | SE | T | P | $P_{rand}$ |
| *Intercept* | 38898 | 2064 | 18.84 | 0.0001 | 0.334 | 38958 | 3802 | 10.25 | 0.0001 | 0.614 |
| *Sex (Male)* | 300 | 1125 | 0.2668 | 0.7908 | 0.784 | 1606 | 2079 | 0.7727 | 0.4442 | 0.468 |
| *Sex (Unk)* | 1439 | 2871 | 0.5011 | 0.6187 | 0.4 | 10075 | 4696 | 2.1456 | 0.0379 | 0.038 |
| *Age (Juv)* | -2302 | 1063 | -2.1646 | 0.0356 | 0.014 | -4876 | 2092 | -2.33 | 0.0248 | 0.028 |
| *Immigrant* | 519 | 1452 | 0.3577 | 0.7222 | 0.62 | 102 | 2753 | 0.037 | 0.9707 | 0.926 |
| *Site* | -2875 | 1696 | -1.6956 | 0.0967 | 0.276 | 1929 | 2575 | 0.7492 | 0.458 | 0.34 |
| *Strength* | -377 | 559 | -0.6739 | 0.5038 | 0.466 | -125 | 1487 | -0.084 | 0.9332 | 0.944 |
| *Observations* | 0.2099 | 0.2216 | 0.9472 | 0.3485 | 0.388 | -1.5294 | 1.4881 | -1.028 | 0.3101 | 0.458 |

**Table S9: Social network strength and overall time delay to use novel food, related to Figure 3.** Output of LMs assessing the relationship between the amount of time taken for each individual to first land on the feeding perch of the novel food (quantified as total elapsed foraging time since they were first detected at the site during the trial – log transformed), and their prior network strength, along with the other fitted explanatory variables. Each column holds the test statistics for (A) experimental trial 1 and (B) experimental trial 2. Each row gives the result for each explanatory variable, with 'Sex' in relation to female birds, Age in relation to adult birds, Immigrant status in relation to residents, and the 'Strength' as weighted network degree directly prior to each experimental trial (see Methods) and 'Observations' as the number of records.

| | (A) Experimental Trial 1 | | | | | (B) Experimental Trial 2 | | | | |
|---|---|---|---|---|---|---|---|---|---|---|
| | Coeff. | SE | T | P | $P_{rand}$ | Coeff. | SE | T | P | $P_{rand}$ |
| *Intercept* | 6.3798 | 1.8885 | 3.3782 | 0.0014 | 0.216 | 2.4033 | 2.3087 | 1.041 | 0.3038 | 0.222 |
| *Sex (Male)* | -0.7159 | 1.101 | -0.6503 | 0.5186 | 0.558 | 1.9838 | 1.2731 | 1.5583 | 0.1267 | 0.136 |
| *Sex (Unk)* | -2.2135 | 2.9084 | -0.7611 | 0.4503 | 0.224 | 4.4115 | 2.9211 | 1.5102 | 0.1385 | 0.076 |
| *Age (Juv)* | -0.4054 | 1.0667 | -0.3801 | 0.7055 | 0.708 | -0.7133 | 1.3014 | -0.5481 | 0.5865 | 0.64 |
| *Immigrant* | -1.1519 | 1.411 | -0.8164 | 0.4182 | 0.42 | 1.3764 | 1.7166 | 0.8018 | 0.4272 | 0.442 |
| *Site* | -0.3937 | 1.5755 | -0.2499 | 0.8037 | 0.028 | 0.1215 | 1.5917 | 0.0764 | 0.9395 | 0.472 |
| *Strength* | -0.7663 | 0.5415 | -1.4151 | 0.1634 | 0.118 | 0.2549 | 0.9233 | 0.276 | 0.7839 | 0.778 |
| *Observations* | 0.0001 | 0.0001 | 3.05 | 0.0037 | 0.002 | 0.0000 | 0.0001 | 0.3954 | 0.6946 | 0.73 |

**Table S10: Social network strength and novel food exploitation after first use, related to Figure 4.** Output of GLMs assessing the relationship between individuals' propensity to use novel food after they had already first tried the novel food feeder (response variable) and individuals' prior network strength (Figure 4 - Main Text), along with the other fitted explanatory variables. Each column holds the test statistics for (A) experimental trial 1 and (B) experimental trial 2. Each row gives the result for each explanatory variable, with 'Sex' in relation to female birds, Age in relation to adult birds, Immigrant status in relation to residents, and the 'Strength' as weighted network degree directly prior to each experimental trial (see Methods) and 'Observations' as the number of records.

| | (A) Experimental Trial 1 | | | | | (B) Experimental Trial 2 | | | | |
|---|---|---|---|---|---|---|---|---|---|---|
| | Coeff. | SE | T | P | $P_{rand}$ | Coeff. | SE | T | P | $P_{rand}$ |
| *Intercept* | -3.5136 | 1.0769 | -3.2627 | 0.002 | 0.001 | 0.4391 | 0.5057 | 0.8682 | 0.3902 | 0.001 |
| *Sex (Male)* | 0.316 | 0.3108 | 1.0167 | 0.3143 | 0.508 | 0.4231 | 0.2235 | 1.8928 | 0.0653 | 0.210 |
| *Sex (Unk)* | -0.1159 | 1.2082 | -0.0959 | 0.924 | 0.886 | -0.0665 | 0.4319 | -0.1539 | 0.8784 | 0.948 |
| *Age (Juv)* | 0.5041 | 0.2892 | 1.7429 | 0.0876 | 0.264 | 0.3009 | 0.2112 | 1.4244 | 0.1617 | 0.386 |
| *Immigrant* | 0.2262 | 0.3783 | 0.598 | 0.5526 | 0.682 | 0.3363 | 0.2452 | 1.3718 | 0.1774 | 0.364 |
| *Site* | 3.6381 | 0.7803 | 4.6627 | 0.0001 | 0.014 | -1.8279 | 0.3112 | -5.8741 | 0.0001 | 0.158 |
| *Strength* | 0.5483 | 0.2551 | 2.1492 | 0.0366 | 0.006 | 0.5324 | 0.1677 | 3.1742 | 0.0028 | 0.010 |
| *Observations* | 0.0001 | 0.0001 | 0.0207 | 0.9836 | 0.994 | 0.0001 | 1E-04 | -0.4635 | 0.6454 | 0.802 |
